# Density Alters Impacts of Genotypic Evenness on Productivity in an Experimental Plant Population

**DOI:** 10.3389/fpls.2022.915812

**Published:** 2022-05-30

**Authors:** Lin Huang, Meng-Fei Yu, Jiang-Nan Hu, Wei-Jia Sheng, Wei Xue, Fei-Hai Yu

**Affiliations:** Zhejiang Provincial Key Laboratory of Evolutionary Ecology and Conservation/Institute of Wetland Ecology & Clone Ecology, Taizhou University, Taizhou, China

**Keywords:** clonal plant, complementarity, functional traits, genotypic richness, genotypic evenness, productivity, selection effects

## Abstract

Genetic diversity plays important roles in maintaining population productivity. While the impact of genotypic richness on productivity has been extensively tested, the role of genotypic evenness has not been considered. Plant density can also affect population productivity, but its interaction with genotypic diversity has not been tested. We constructed experimental populations of the clonal plant *Hydrocotyle vulgaris* with either low or high richness (consisting of four vs. eight genotypes), either low or high evenness (each genotype had a different number vs. the same number of ramets), and either low or high density (consisting of 16 vs. 32 ramets) in a full factorial design. Total biomass of plant populations did not differ between four- and eight-genotype mixtures. When the initial plant density was low, total biomass of populations with high genotypic evenness was significantly greater than total biomass of those with low genotypic evenness. However, this difference disappeared when the initial plant density was high. Moreover, total biomass increased linearly with increasing plant density at harvest, but was negatively correlated to variation in leaf area. We conclude that genotypic evenness but not genotypic richness can benefit population productivity, and that plant density can alter the impact of genotypic evenness on population productivity.

## Introduction

A large body of studies have investigated the relationship between plant diversity and ecosystem processes, such as primary productivity (e.g., Tilman et al., 2001; van Ruijven and Berendse, 2005; Hector, 2011), decomposition (e.g., Gessner et al., 2010; Mori et al., 2020), invasion resistance (e.g., van Ruijven et al., 2003; Fargione and Tilman, 2005), and ecosystem stability (e.g., Tilman et al., 2006; Isbell et al., 2015). These studies revealed worldwide a positive relationship between plant species diversity and ecosystem functioning (Isbell et al., 2011; Liang et al., 2016; Jochum et al., 2020). The mechanisms behind these positive diversity effects were generally attributed to a more complementary utilization of resources (i.e., complementary effect, for example, Loreau and Hector, 2001; van Ruijven and Berendse, 2003) *via* niche differentiation and species facilitation, and/or the presence of dominantly productive species (i.e., sampling effect; Polley et al., 2003; Godoy et al., 2020). However, most of these studies have ignored the potential effects of genotypic diversity.

As the key component of biodiversity, plant genetic diversity also has important ecological consequences on ecosystem functions and services (Hughes et al., 2008). Different genotypes of the same species vary greatly in their ways and abilities to acquire essential resources (e.g., Lankau and Strauss, 2007; Fridley and Grime, 2010; Baron et al., 2015; Zhang et al., 2022). Therefore, increasing genetic diversity may also promote the complementary utilization of resources and increase probability of the presence of particularly productive genotypes, which may eventually increase the primary productivity of plant populations and communities (Lankau and Strauss, 2007; Fridley and Grime, 2010; Schöb et al., 2015). Many studies have indeed found that increasing genetic diversity increases plant primary productivity (Hughes and Stachowicz, 2004; Crutsinger et al., 2006; Lankau and Strauss, 2007; Kotowska et al., 2010), despite that the neutral genetic diversity effect has also been reported (Fridley and Grime, 2010; Prieto et al., 2015; Schöb et al., 2015). However, these few studies have consistently investigated the effect of genetic richness, that is, the number of genotypes on productivity, but have not tested how genetic evenness, that is, the relative contribution of each genotype may influence plant primary productivity.

The importance of species evenness in influencing primary productivity of plant communities has been well documented (Nijs and Roy, 2000; Hillebrand et al., 2008; Orwin et al., 2014; LaManna et al., 2017). A decline in species evenness (or an increase in species dominance) may reduce complementary utilization of resources or species facilitation, and thus plant community productivity (Polley et al., 2003; Kirwan et al., 2007; Hillebrand et al., 2008). However, a decline in species evenness (or an increase in species dominance) may also enhance plant community productivity if the most productive species dominates the community (Nijs and Roy, 2000; Polley et al., 2003; Hillebrand et al., 2008). Similarly, the effects of genotypic evenness on population productivity may also vary depending on the relative importance of complementarity and selection effects among different genotypes. A negative effect of genotypic evenness on population productivity will occur if the complementary resources utilization or facilitations are limited; while a positive effect of genotypic evenness will occur if the most productive genotypes dominate the population.

The diversity effects on plant primary productivity were generally tested in manipulated biodiversity experiments where the total density was kept constant (reviewed in Fraser et al., 2015). However, plant density may potentially influence diversity effects (Wilsey and Stirling, 2007). Increasing plant density may reduce diversity effects as it can compensate for the low productivity at low diversity and thus flatter the diversity–productivity relationship (He et al., 2005). By contrast, many studies found that increasing plant density can enhance diversity effects on productivity, due to enhanced complementarity or selection effect (e.g., Marquard et al., 2009; Stachová et al., 2012). However, plant density may have little influences on diversity effects when the constant final yield is achieved (Weiner and Freckleton, 2010). So far, most of the direct evidence for influences of plant density on diversity effects came from manipulated studies at the species level, whether plant density influences genotypic diversity effects is largely unknown.

To examine the influences of plant density on genotypic diversity effects, we conducted a full factorial greenhouse experiment by manipulating plant density, genotypic richness, and genotypic evenness simultaneously. We mainly tested the following hypothesis: (1) plant population with more genotypes produces larger biomass than that with less genotypes; (2) Plant population with even distribution of genotype abundance produces larger biomass than that with uneven distribution of genotype abundance. (3) Initial plant density alters biomass responses to the number and abundance distribution of genotypes.

## Materials and Methods

### The Species

We used a creeping stem clonal plant *Hydrocotyle vulgaris* L. (Araliaceae) in this experiment. This species has been introduced into China for more than 30 years. It is now widely distributed in many habitats, such as blogs, wetlands, valleys, dunes, and marshes. This species can produce ramets (consisting of a leaf and some adventitious roots) connected by stolon-like creeping stems, that is, internodes (Dong, 1995; Xue et al., 2022). *H. vulgaris* is considered potentially invasive in China as it can exclude other native species due to high phenotypic plasticity and rapidly vegetative growth (Dong et al., 2015). Therefore, to explore the population growth of *H. vulgaris* regulated by density, genotypic richness, and genotypic evenness may have significant implications for the control of its invasion.

We used ten genotypes of *H. vulgaris* in this experiment. The initial ramets of *H. vulgaris* were collected from five Provinces in China in 2016, and their genotypes were identified using methylation-sensitive amplified polymorphism (MSAP) markers (see Wang et al., 2020 for more details). In brief, *H. vulgaris* samples were collected from filed populations, and total genomic DNA was extracted from the mature leaves of the collected plants, then the DNA methylation status of the samples were detected using MSAP. These plants with different DNA methylation status were treated as different genotypes and cultivated in separate containers. Ramets were collected from these containers and used in the experiment described below. Each collected ramet had a node with some adventitious roots, a petiole of 2 cm long, a proximal, and a distal internode of 1 cm long.

### The Experiment

The experiment consisted of two levels of genotypic richness (4 vs. 8 genotypes) and two levels of genotypic evenness (low vs. high evenness), crossed with two levels of plant density (16 vs. 32 ramets). For each level of the genotypic richness treatment, we randomly selected ten genotype combinations with the constraint that each genotype was equally represented (i.e., every genotype presented at the same number of times at each genotypic richness level). For example, for the 4-genotype treatment, a total of $C_{10}^{4}=210$ combinations can be generated if any four out of the 10 genotypes were randomly selected. However, we only selected 10 of these combinations, and for the selected 10 combinations, the occurrence frequency of each genotype was equal. Therefore, in this experiment, we manipulated the number of genotypes rather the genotypic composition; the ten randomly selected combinations for a given treatment can be treated as ten replicates.

We altered the ramet number of each component genotype to create the two evenness and density treatments. For the high evenness treatment, an equal number of ramets was assigned to each component genotype. Therefore, the number of each component genotype was manipulated as 4:4:4:4 (16 ramets in total) or 8:8:8:8 (32 ramets in total) for the 4-genotype combinations, and 2:2:2:2:2:2:2:2 (16 ramets in total) or 4:4:4:4:4:4:4:4 (32 ramets in total) for the 8-genotype combinations. For the low evenness treatment, the ramet number of each component genotype was manipulated as 10:3:2:1 (16 ramets in total) or 20:6:4:2 (32 ramets in total) for the 4-genotype combinations, and 9:1:1:1:1:1:1:1 (16 ramets in total) or 18:2:2:2:2:2:2:2 (32 ramets in total) for the 8-genotype combinations. Each genotype was assigned equally to different proportions. The initial Pielou’s evenness index (calculated as: $H^{\prime }/LnS$, where *H′* is the Shannon–Wiener diversity index, and *S* is the number of genotypes) in the high evenness treatment was equal to one; but that in the low evenness treatment were 0.75 and 0.74 for the 4- and 8-genotype mixtures, respectively. Therefore, there were 2 genotypic richness × 2 genotypic evenness × 2 density × 10 combinations (replicates) = 80 pots in total.

**Figure 1:**
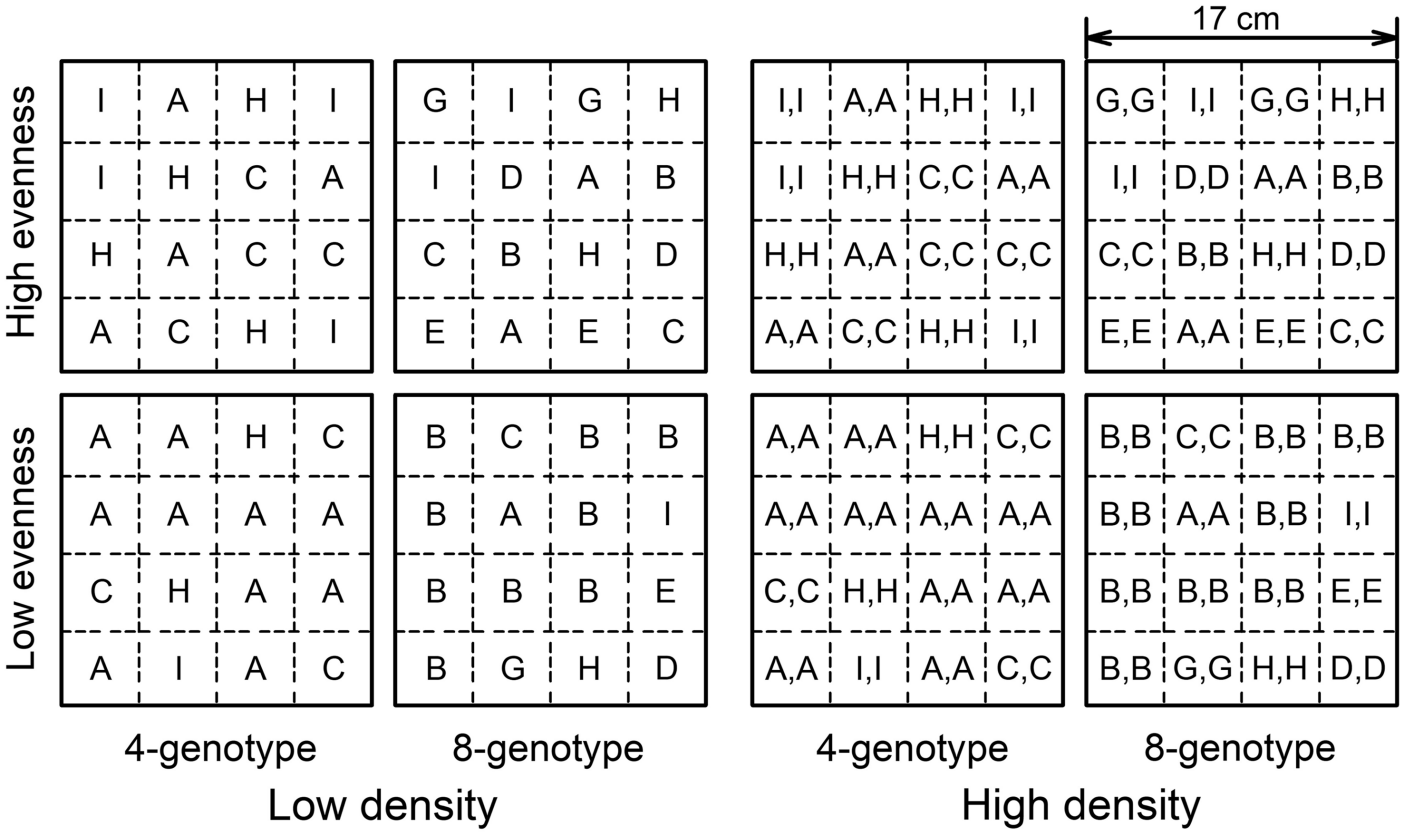
Schematic representation of the experimental design. The experiment consisted of two levels of genotypic richness treatment (4-genotype vs. 8-genotype), two levels of genotypic evenness treatment (low evenness vs. high evenness), fully crossed with two levels of plant density treatment (low density vs. high density). 16 ramets (low density) or 32 ramets (high density) from four (low richness) or eight genotypes (high richness) were planted in a pot with the component genotypes had an equal abundance (high evenness) or not (low evenness). Different letters (A-I) represent ramets of different genotypes and mark the initial planting positions (presented are the genotype composition and planting patterns in one of the ten randomly selected compositions).

Before planting, we filled each pot (17 cm × 17 cm × 11 cm) with potting soil (total nitrogen: 0.17 g kg^−1^, total phosphorus: 0.002 g kg^−1^, total potassium 1.5 g kg^−1^; Hebei Dewoduo Fertilizer Co., Ltd., Hengshui, China). We then divided the soil surface into 16 equivalent patches and planted ramets of the correspondent genotypes in these patches; each patch had one (16 ramets in total) or two ramets (32 ramets in total) of the same genotype (see Figure 1 for an example).

All pots were watered regularly. The experiment was maintained for 65 days (30 May to 5 August 2020). During the experiment, the daily mean temperature was 30.2°C.

### Harvest and Measurements

At the end of the experiment, it is not possible to distinguish the ramets of different genotypes in a pot (population). Therefore, we first counted the number of all ramets in each pot. Then, for each pot, we harvested ramets of different genotypes together. All the ramets in a pot were separated into roots, creeping stems, and leaves; dry weight of the roots, creeping stems and leaves were the root biomass, creeping stem biomass, and leave biomass of the population, respectively. The sum of the root biomass, creeping stem biomass, and leave biomass was the total biomass (i.e., productivity) of the population. During harvest, the roots were washed over a 0.5-mm-mesh sieve. We also randomly selected five fully developed leaf blades in each pot and measured leaf area and dry weight of these five selected leaves. Based on these data, we calculated variation in leaf area and specific leaf area (i.e., ratio of mean leaf area to mean dry weight, SLA). We also measured the total creeping stem length in each pot and counted the number of internode. Based on these data, we calculated the internode length as the ratio of total creeping stem length to number of internode. The dry weight of all plant materials was obtained after being oven-dried at 70°C for at least 48 h.

### Data Analysis

We used three-way ANOVAs to examine the effect of genotypic richness, genotypic evenness, plant density, and their interactions on the growth (i.e., total biomass, root biomass, creeping stem biomass, leaf biomass, and number of ramets) and morphological traits (i.e., internode length, variation in leaf area, and SLA) of the population. When a significant effect was detected, post-hoc *Duncan’s* test were used to compare means.

To explore the underlying mechanisms, we further tested the relationship between total biomass and number of ramets, a trait associated with life history. We also tested the relationship between total biomass and morphological traits, that is, internode length, variation in leaf area, and specific leaf area that associated with light capture ability. These relationships were examined separately in a simple linear regression.

All analyses were performed in SPSS (version 22). Residuals of all variables were checked for normality and homogeneity of variance using Shapiro–Wilk test and Levene’s test, respectively.

## Results

### Population Growth

**Figure 2:**
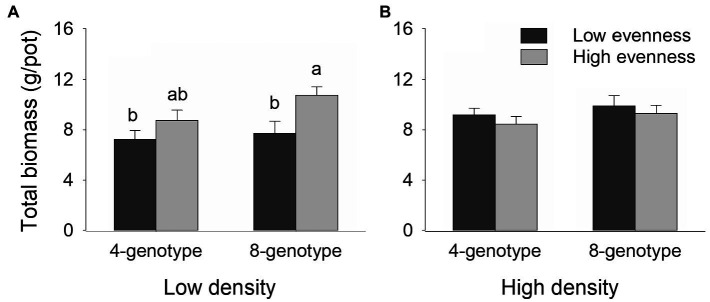
Effects of genotypic richness (4-gneotype vs. 8-genotype), genotypic evenness (low vs. high evenness), and plant density (low vs. high density) on total biomass **(A,B)** of *Hydrocotyle vulgaris* population. Mean values (+1 SE) are presented; see Table 1 for statistic results. Different letters (a–b) at the end of bars indicate significant difference in each panel.

**Figure 3:**
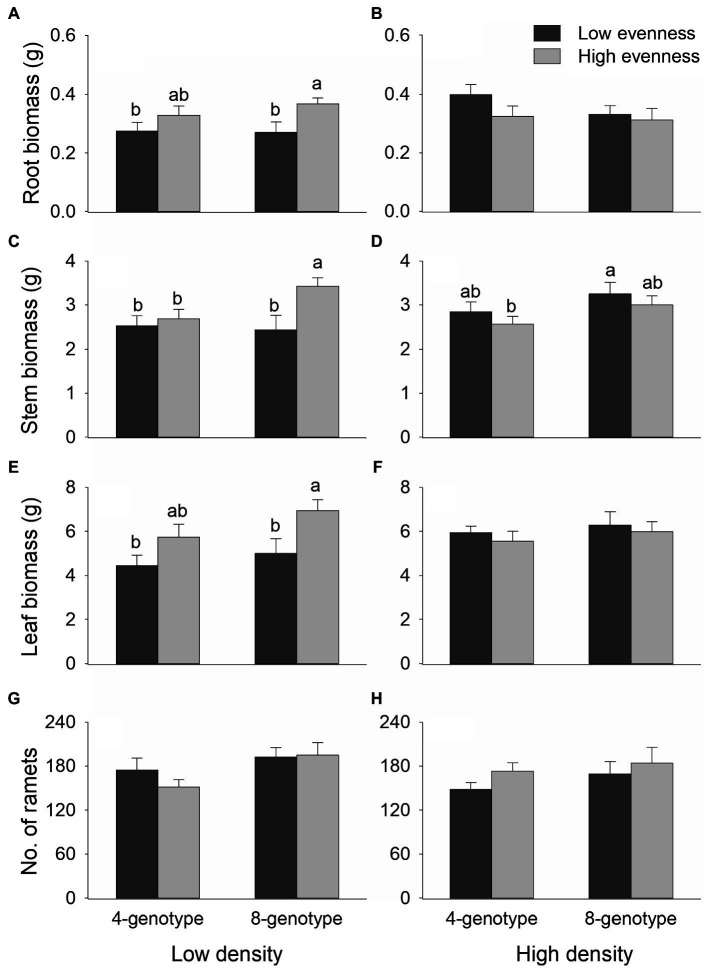
Effects of genotypic richness (4-gneotype vs. 8-genotype), genotypic evenness (low vs. high evenness), and plant density (low vs. high density) on root biomass **(A,B)**, stem biomass **(C,D)**, leaf biomass **(E,F)** and ramet number **(G,H)** of *Hydrocotyle vulgaris* population. Mean values (+1 SE) are presented; see Table 1 for statistic results. Different letters (a–b) at the end of bars indicate significant difference in each panel.

Total biomass, root biomass, and leaf biomass of *H. vulgaris* population were not significantly different in the four- and eight-genotype mixtures (Figures 2, 3A,B,E,F; Table 1). However, stem biomass and ramet number of *H. vulgaris* population were significantly greater in eight-genotype mixtures than in four-genotype mixtures (Figures 3C,D,G,H; Table 1). When the initial plant density was low, all biomass measures of *H. vulgaris* population with high genotypic evenness were significantly greater than that with low genotypic evenness (Figures 2A, 3A,C,E; Table 1). This difference, however, disappeared when the initial plant density was high (Figures 2B, 3B,D,F; Table 1).

**Table 1:** Effects of genotypic richness (4-genotype vs. 8-genotype), genotypic evenness (low vs. high evenness), and plant density (low vs. high density) on total biomass, root biomass, creeping stem biomass, leaf biomass, number of ramets, internode length, variation in leaf area, and specific leaf area (SLA) of *Hydrocotyle vulgaris* population.

Effect	Total biomass		No. of ramets		Root biomass		Stem biomass		Leaf biomass		Internode length^1^		Variation in leaf area		SLA	
	*F*	*P*	*F*	*P*	*F*	*P*	*F*	*P*	*F*	*P*	*F*	*P*	*F*	*P*	*F*	*P*
Richness (R)	3.72	*0.058*	4.92	**0.030**	0.23	0.636	5.13	**0.027**	2.98	*0.089*	6.39	**0.014**	0.26	0.613	0.86	0.358
Evenness (E)	2.41	0.125	0.20	0.660	0.42	0.518	0.86	0.358	2.99	*0.088*	<0.01	0.975	4.71	**0.033**	0.53	0.469
Density (D)	1.28	0.261	0.84	0.361	1.74	0.191	0.78	0.379	1.24	0.270	0.19	0.666	1.40	0.241	0.40	0.528
R × E	0.68	0.414	0.12	0.726	1.12	0.293	1.68	0.200	0.26	0.613	0.71	0.403	1.13	0.291	0.89	0.350
R × D	0.19	0.663	0.49	0.488	1.57	0.214	0.08	0.785	0.44	0.511	0.45	0.505	1.28	0.262	1.01	0.318
E × D	7.95	**0.006**	1.93	0.169	6.96	**0.010**	6.43	**0.013**	7.08	**0.010**	0.44	0.510	<0.01	0.985	0.14	0.709
R × E × D	0.44	0.509	0.71	0.402	0.02	0.880	1.50	0.224	0.15	0.696	2.52	0.117	0.81	0.372	0.76	0.385

1 Data were ln-transformed. Values are in bold when p < 0.05, and in italic when p < 0.1. Degree of freedom is 1, 72 for all effects.

### Population Morphological Traits

**Figure 4:**
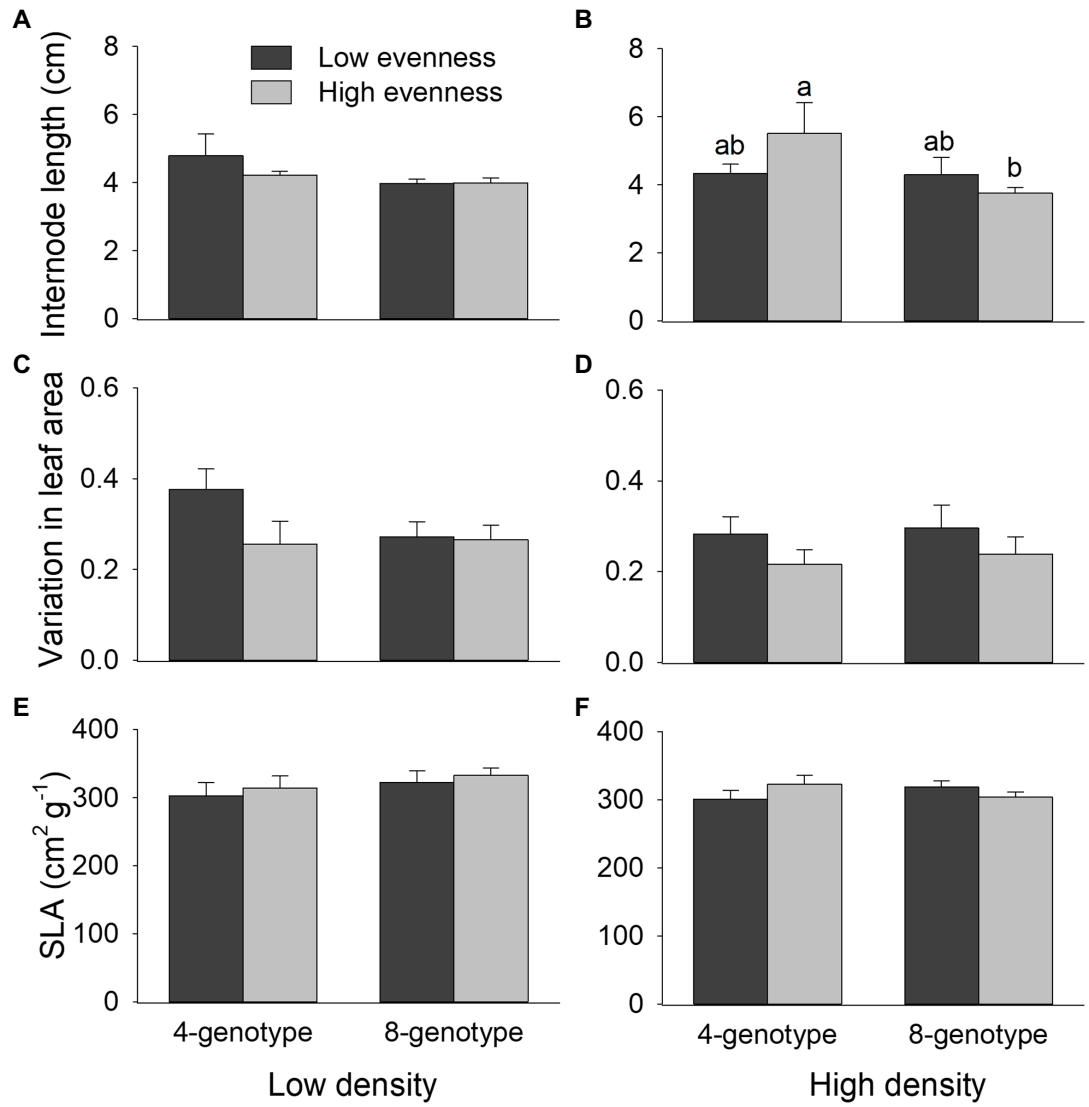
Effects of genotypic richness (4-gneotype vs. 8-genotype), genotypic evenness (low vs. high evenness), and plant density (low vs. high density) on internode length **(A,B)**, variation in leaf area **(C,D)**, and specific leaf area (SLA; **E,F**) of *Hydrocotyle vulgaris* population. Mean values (+1 SE) are presented; see Table 1 for statistic results. Different letters (a–b) at the end of bars indicate significant difference in each panel.

Internode length of *H. vulgaris* population was overall greater in four-genotype mixtures than in eight-genotype mixtures, but genotypic evenness or plant density did not influence internode length of the population (Figures 4A,B; Table 1). Variation in leaf area was overall greater in *H. vulgaris* population with low genotypic evenness than that with high genotypic evenness, but genotypic richness or plant density had no effects on variation in leaf area (Figures 4C,D; Table 1). Genotypic richness, genotypic evenness, plant density, or their interactions did not influence specific leaf area (Figures 4E,F; Table 1).

### Relationship Between Population Productivity and Morphological Traits

**Figure 5:**
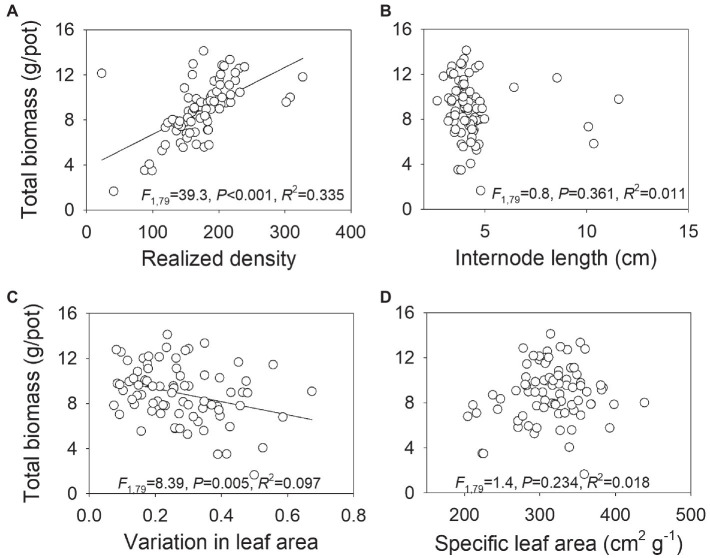
Relationship between total biomass and realized density (i.e., number of ramets) at harvest **(A)**, internode length **(B)**, variation in leaf area **(C)**, and specific leaf area **(D)** in *Hydrocotyle vulgaris* population. Each dot represents a data point for a population. *F*-, *R*^2^-, and *p*-values obtained from linear regressions are also presented.

There was a significantly positive relationship between total biomass and number of ramets at harvest (Figure 5A), indicating that the density employed in our study was not sufficient to reach constant final yield. However, total biomass was significantly negatively correlated to variation in leaf area (Figure 5C). No significant relationship was found between total biomass and internode length or specific leaf area (Figures 5B,D).

## Discussions

In this study, we showed an overall positive genotypic evenness effect but a neutral genotypic richness effect on productivity in *H. vulgaris* population. Moreover, the effect of genotypic evenness was only observed when the plant density was relatively low. These results indicate that, plant density may alter the relationship between genotypic diversity and population productivity through changing the effect of genotypic evenness rather than that of genotypic richness.

The few studies invested influences of genotypic richness on plant productivity found both neutral (Fridley and Grime, 2010; Prieto et al., 2015; Schöb et al., 2015) and positive effects (Crutsinger et al., 2006; Kotowska et al., 2010). Here we also found a neutral effect of genotypic richness in *H. vulgaris*. However, we found that genotypic evenness can significantly increase productivity of *H. vulgaris* population of low density. Therefore, these results indicate that genotypic evenness rather than genotypic richness may influence productivity of *H. vulgaris* population. Complementary effects and sampling effects, as shown in many biodiversity studies at the species level (Loreau and Hector, 2001; van Ruijven and Berendse, 2003; Gross et al., 2007; Hector, 2011; Zuppinger-Dingley et al., 2014), may also explain the positive genotypic diversity effects in our study. Unfortunately, our experiment design prevented us separating these two effects directly. However, we found a significant negative relationship between population productivity and variation in leaf area which is one of the key traits associated with light capture ability. This result indicates that selection effects in the utilization of light resources may have played a major role in the positive genotypic diversity effect (Brett Mattingly et al., 2007).

Our results also showed that genotypic evenness may be more important than genotypic richness in influencing population productivity of *H. vulgaris*. This is likely because it is difficult to detect a difference in genotypic richness in a short-term greenhouse experiment as it may take a very long time for a particular genotype to disappear at the local population. However, the relative abundance of the genotype may vary during a relatively short time period as it is much more sensitive to local plant–plant interactions (Mulder et al., 2004). Therefore, it is urgently needed to detect the relative importance of genotypic richness and genotypic evenness in driving plant primary productivity in long-term manipulated experiments.

As expected, plant density altered the effect of genotypic evenness on primary productivity of population *H. vulgaris*. In our study, total biomass of plant population showed a linear increase with plant density (i.e., number of ramets) at harvest, indicating that the density employed in our study was not sufficient to reach constant final yield where the production does not increase with density further (Weiner and Freckleton, 2010). Therefore, increasing plant density may have potentially compensated for the low productivity in the low evenness treatment (as shown in Figure 2) in which the competition between different genotypes was intense. This process may have eventually led to the observed neutral genotypic evenness effect in the high-density treatment.

By contrast, we did not find the evidence that plant density influences the effect of genotypic richness on primary productivity of *H. vulgaris*, a similar result has also been reported in *Arabidopsis thaliana* (Kotowska et al., 2010). However, the underlying mechanism was not clear. In many species-level studies manipulating both plant density and species richness, plant density was found to alter the effect of species richness on community productivity through changing complementarity or selection effects (e.g., Marquard et al., 2009; Stachová et al., 2012; Shovon et al., 2022). Therefore, as has been declared in a species-level study (Schmitz et al., 2013), the absence of density impacts on the genotypic richness effects is likely because the difference in the strength of complementarity or selection effects between mixtures of low and high genotypic richness was consistent in the low- and high-density treatment.

In conclusion, genotypic evenness rather than genotypic richness can influence productivity of *H. vulgaris* population. Moreover, the effect of genotypic evenness varied depending on plant density. These results indicate that genotypic evenness may be more important than genotypic richness in mediating plant population productivity. However, it should be noted that these effects may change as the development of plant populations and vary between different plant populations. Therefore, further studies should focus on dynamic changes of the joint effect of genotypic richness, genotypic evenness, and plant density by including multiple plant populations.

## Data Availability Statement

The original contributions presented in the study are included in the article/supplementary material, further inquiries can be directed to the corresponding authors.

## Author Contributions

LH, WX, and F-HY designed the study. M-FY, J-NH, and W-JS conducted the experiment and collected the data. LH and WX analyzed the data. LH, WX, and F-HY wrote the first version of the manuscript. All authors contributed to the article and approved the submitted version.

## Funding

This research was financially supported by National Natural Science Foundation of China (grant 32001122) and Zhejiang Provincial Natural Science Foundation (grant LQ21C030003).

## Conflict of Interest

The authors declare that the research was conducted in the absence of any commercial or financial relationships that could be construed as a potential conflict of interest.

## Publisher’s Note

All claims expressed in this article are solely those of the authors and do not necessarily represent those of their affiliated organizations, or those of the publisher, the editors and the reviewers. Any product that may be evaluated in this article, or claim that may be made by its manufacturer, is not guaranteed or endorsed by the publisher.

## References

[ref1] Baron E. Richirt J. Villoutreix R. Amsellem L. Roux F. (2015). The genetics of intra- and interspecific competitive response and effect in a local population of an annual plant species. Funct. Ecol. 29, 1361–1370. doi: 10.1111/1365-2435.12436

[ref2] Brett Mattingly W. Hewlate R. Reynolds H. L. (2007). Species evenness and invasion resistance of experimental grassland communities. Oikos 116, 1164–1170. doi: 10.1111/j.0030-1299.2007.15406.x

[ref3] Crutsinger G. M. Collins M. D. Fordyce J. A. Gompert Z. Nice C. C. Sanders N. J. (2006). Plant genotypic diversity predicts community structure and governs an ecosystem process. Science 313, 966–968. doi: 10.1126/science.1128326, PMID: 16917062

[ref4] Dong M. (1995). Morphological responses to local light conditions in clonal herbs from contrasting habitats, and their modification due to physiological integration. Oecologia 101, 282–288. doi: 10.1007/BF00328813, PMID: 28307048

[ref5] Dong B.-C. Wang J.-Z. Liu R.-H. Zhang M.-X. Luo F.-L. Yu F.-H. (2015). Soil heterogeneity affects ramet placement of *Hydrocotyle vulgaris*. J. Plant Ecol. 8, 91–100. doi: 10.1093/jpe/rtu003

[ref6] Fargione J. E. Tilman D. (2005). Diversity decreases invasion via both sampling and complementarity effects. Ecol. Lett. 8, 604–611. doi: 10.1111/j.1461-0248.2005.00753.x

[ref7] Fraser L. H. Pither J. Jentsch A. Sternberg M. Zobel M. Askarizadeh D.. (2015). Worldwide evidence of a unimodal relationship between productivity and plant species richness. Science 349, 302–305. doi: 10.1126/science.aab3916, PMID: 26185249

[ref8] Fridley J. D. Grime J. P. (2010). Community and ecosystem effects of intraspecific genetic diversity in grassland microcosms of varying species diversity. Ecology 91, 2272–2283. doi: 10.1890/09-1240.1, PMID: 20836449

[ref9] Gessner M. O. Swan C. M. Dang C. K. McKie B. G. Bardgett R. D. Wall D. H.. (2010). Diversity meets decomposition. Trends Ecol. Evol. 25, 372–380. doi: 10.1016/j.tree.2010.01.010, PMID: 20189677

[ref10] Godoy O. Gómez-Aparicio L. Matías L. Pérez-Ramos I. M. Allan E. (2020). An excess of niche differences maximizes ecosystem functioning. Nat. Commun. 11:4180. doi: 10.1038/s41467-020-17960-5, PMID: 32826915 PMC7442808

[ref11] Gross N. Suding K. N. Lavorel S. Roumet C. (2007). Complementarity as a mechanism of coexistence between functional groups of grasses. J. Ecol. 95, 1296–1305. doi: 10.1111/j.1365-2745.2007.01303.x

[ref12] He J.-S. Wolfe-Bellin K. S. Schmid B. Bazzaz F. A. (2005). Density may alter diversity–productivity relationships in experimental plant communities. Basic Appl. Ecol. 6, 505–517. doi: 10.1016/j.baae.2005.04.002

[ref13] Hector A. (2011). Diversity favours productivity. Nature 472, 45–46. doi: 10.1038/472045a, PMID: 21475190

[ref14] Hillebrand H. Bennett D. M. Cadotte M. W. (2008). Consequences of dominance: a review of evenness effects on local and regional ecosystem processes. Ecology 89, 1510–1520. doi: 10.1890/07-1053.1, PMID: 18589516

[ref15] Hughes A. R. Inouye B. D. Johnson M. T. J. Underwood N. Vellend M. (2008). Ecological consequences of genetic diversity. Ecol. Lett. 11, 609–623. doi: 10.1111/j.1461-0248.2008.01179.x 18400018

[ref16] Hughes A. R. Stachowicz J. J. (2004). Genetic diversity enhances the resistance of a seagrass ecosystem to disturbance. Proc. Natl. Acad. Sci. USA 101, 8998–9002. doi: 10.1073/pnas.0402642101, PMID: 15184681 PMC428461

[ref17] Isbell F. Calcagno V. Hector A. Connolly J. Harpole W. S. Reich P. B.. (2011). High plant diversity is needed to maintain ecosystem services. Nature 477, 199–202. doi: 10.1038/nature10282, PMID: 21832994

[ref18] Isbell F. Craven D. Connolly J. Loreau M. Schmid B. Beierkuhnlein C.. (2015). Biodiversity increases the resistance of ecosystem productivity to climate extremes. Nature 526, 574–577. doi: 10.1038/nature15374, PMID: 26466564

[ref19] Jochum M. Fischer M. Isbell F. Roscher C. van der Plas F. Boch S.. (2020). The results of biodiversity–ecosystem functioning experiments are realistic. Nat. Ecol. Evol. 4, 1485–1494. doi: 10.1038/s41559-020-1280-9, PMID: 32839545

[ref20] Kirwan L. LÜScher A. Sebasti À. M. T. Finn J. A. Collins R. P. Porqueddu C.. (2007). Evenness drives consistent diversity effects in intensive grassland systems across 28 European sites. J. Ecol. 95, 530–539. doi: 10.1111/j.1365-2745.2007.01225.x

[ref21] Kotowska A. M. Cahill J. F. Jr. Keddie B. A. (2010). Plant genetic diversity yields increased plant productivity and herbivore performance. J. Ecol. 98, 237–245. doi: 10.1111/j.1365-2745.2009.01606.x

[ref22] LaManna J. A. Belote R. T. Burkle L. A. Catano C. P. Myers J. A. (2017). Negative density dependence mediates biodiversity–productivity relationships across scales. Nat. Ecol. Evol. 1, 1107–1115. doi: 10.1038/s41559-017-0225-4, PMID: 29046568

[ref23] Lankau R. A. Strauss S. Y. (2007). Mutual feedbacks maintain both genetic and species diversity in a plant community. Science 317, 1561–1563. doi: 10.1126/science.1147455, PMID: 17872447

[ref24] Liang J. Crowther T. W. Picard N. Wiser S. Zhou M. Alberti G.. (2016). Positive biodiversity-productivity relationship predominant in global forests. Science 354:aaf8957. doi: 10.1126/science.aaf8957, PMID: 27738143

[ref25] Loreau M. Hector A. (2001). Partitioning selection and complementarity in biodiversity experiments. Nature 412, 72–76. doi: 10.1038/35083573 11452308

[ref26] Marquard E. Weigelt A. Roscher C. Gubsch M. Lipowsky A. Schmid B. (2009). Positive biodiversity–productivity relationship due to increased plant density. J. Ecol. 97, 696–704. doi: 10.1111/j.1365-2745.2009.01521.x

[ref27] Mori A. S. Cornelissen J. H. C. Fujii S. Okada K. I. Isbell F. (2020). A meta-analysis on decomposition quantifies afterlife effects of plant diversity as a global change driver. Nat. Commun. 11:4547. doi: 10.1038/s41467-020-18296-w, PMID: 32917880 PMC7486393

[ref28] Mulder C. P. H. Bazeley-White E. Dimitrakopoulos P. G. Hector A. Scherer-Lorenzen M. Schmid B. (2004). Species evenness and productivity in experimental plant communities. Oikos 107, 50–63. doi: 10.1111/j.0030-1299.2004.13110.x

[ref29] Nijs I. Roy J. (2000). How important are species richness, species evenness and interspecific differences to productivity? A mathematical model. Oikos 88, 57–66. doi: 10.1034/j.1600-0706.2000.880107.x

[ref30] Orwin K. H. Ostle N. Wilby A. Bardgett R. D. (2014). Effects of species evenness and dominant species identity on multiple ecosystem functions in model grassland communities. Oecologia 174, 979–992. doi: 10.1007/s00442-013-2814-5, PMID: 24213721

[ref31] Polley H. W. Wilsey B. J. Derner J. D. (2003). Do species evenness and plant density influence the magnitude of selection and complementarity effects in annual plant species mixtures? Ecol. Lett. 6, 248–256. doi: 10.1046/j.1461-0248.2003.00422.x

[ref32] Prieto I. Violle C. Barre P. Durand J.-L. Ghesquiere M. Litrico I. (2015). Complementary effects of species and genetic diversity on productivity and stability of sown grasslands. Nat. Plants 1, 1–5. doi: 10.1038/nplants.2015.33 27247033

[ref33] Schmitz M. Flynn D. F. B. Mwangi P. N. Schmid R. Scherer-Lorenzen M. Weisser W. W.. (2013). Consistent effects of biodiversity on ecosystem functioning under varying density and evenness. Folia Geobot. 48, 335–353. doi: 10.1007/s12224-013-9177-x

[ref34] Schöb C. Kerle S. Karley A. J. Morcillo L. Pakeman R. J. Newton A. C.. (2015). Intraspecific genetic diversity and composition modify species-level diversity–productivity relationships. New Phytol. 205, 720–730. doi: 10.1111/nph.13043, PMID: 25250812

[ref35] Shovon T. A. Kang S. Scherer-Lorenzen M. Nock C. A. (2022). Changes in the direction of the diversity–productivity relationship over 15 years of stand development in a planted temperate forest. J. Ecol. 110, 1125–1137. doi: 10.1111/1365-2745.13855

[ref36] Stachová T. Fibich P. Lepš J. (2012). Plant density affects measures of biodiversity effects. J. Plant Ecol. 6, 1–11. doi: 10.1093/jpe/rts015

[ref37] Tilman D. Reich P. B. Knops J. M. (2006). Biodiversity and ecosystem stability in a decade-long grassland experiment. Nature 441, 629–632. doi: 10.1038/nature04742, PMID: 16738658

[ref38] Tilman D. Reich P. B. Knops J. Wedin D. Mielke T. Lehman C. (2001). Diversity and productivity in a long-term grassland experiment. Science 294, 843–845. doi: 10.1126/science.1060391, PMID: 11679667

[ref39] van Ruijven J. Berendse F. (2003). Positive effects of plant species diversity on productivity in the absence of legumes. Ecol. Lett. 6, 170–175. doi: 10.1046/j.1461-0248.2003.00427.x

[ref40] van Ruijven J. Berendse F. (2005). Diversity–productivity relationships: initial effects, long-term patterns, and underlying mechanisms. P. Natl. Acad. Sci. USA 102, 695–700. doi: 10.1073/pnas.0407524102, PMID: 15640357 PMC545547

[ref41] van Ruijven J. De Deyn G. B. Berendse F. (2003). Diversity reduces invasibility in experimental plant communities: the role of plant species. Ecol. Lett. 6, 910–918. doi: 10.1046/j.1461-0248.2003.00516.x

[ref42] Wang M.-Z. Li H.-L. Li J.-M. Yu F.-H. (2020). Correlations between genetic, epigenetic and phenotypic variation of an introduced clonal herb. Heredity 124, 146–155. doi: 10.1038/s41437-019-0261-8, PMID: 31431739 PMC6906319

[ref43] Weiner J. Freckleton R. P. (2010). Constant final yield. Annu. Rev. Ecol. Evol. S. 41, 173–192. doi: 10.1146/annurev-ecolsys-102209-144642

[ref44] Wilsey B. Stirling G. (2007). Species richness and evenness respond in a different manner to propagule density in developing prairie microcosm communities. Plant Ecol. 190, 259–273. doi: 10.1007/s11258-006-9206-4

[ref45] Xue W. Huang L. Sheng W.-J. Zhu J.-T. Li S.-Q. Yu F.-H. (2022). Contrasting effects of plant-soil feedbacks on growth and morphology of physically-connected daughter and mother ramets in two clonal plants. Plant Soil 472, 479–489. doi: 10.1007/s11104-021-05266-4

[ref46] Zhang L.-M. Roiloa S. R. Xue W. Yu F.-H. (2022). Effects of temporal heterogeneity in nutrient supply on intra- and inter-genet competition of a clonal herb. Glob. Ecol. Conserv. 35:e02076. doi: 10.1016/j.gecco.2022.e02076

[ref47] Zuppinger-Dingley D. Schmid B. Petermann J. S. Yadav V. Deyn G. B. D. Flynn D. F. B. (2014). Selection for niche differentiation in plant communities increases biodiversity effects. Nature 515, 108–111. doi: 10.1038/nature13869, PMID: 25317555

